# Estimating HIV incidence from surveillance data indicates a second wave of infections in Brazil

**DOI:** 10.1016/j.epidem.2019.02.002

**Published:** 2019-06

**Authors:** Tara D. Mangal, Ana Roberta Pati Pascom, Juan F. Vesga, Mariana Veloso Meireles, Adele Schwartz Benzaken, Timothy B. Hallett

**Affiliations:** a Medical Research Council Centre for Outbreak Analysis and Modelling, Department of Infectious Disease Epidemiology, School of Public Health, Imperial College London, Norfolk Place, London, W2 1PG, United Kingdom; b Ministry of Health, Department of STI, HIV/AIDS and Viral Hepatitis, SRTVN Quadra 701, Lote D, Edifício PO700, CEP: 70719-040, Brasilia, Distrito Federal, Brazil

**Keywords:** HIV/AIDS, Incidence estimation, Brazil, Deterministic model, Prevalence, HIV care cascade

## Abstract

Emerging evidence suggests that HIV incidence rates in Brazil, particularly among men, may be rising. Here we use Brazil’s integrated health systems data to develop a mathematical model, reproducing the complex surveillance systems and providing estimates of HIV incidence, number of people living with HIV (PLHIV), reporting rates and ART initiation rates.

An age-structured deterministic model with a flexible spline was used to describe the natural history of HIV along with reporting and treatment rates. Individual-level surveillance data for 1,077,295 cases (HIV/AIDS diagnoses, ART dispensations, CD4 counts and HIV/AIDS-related deaths) were used to calibrate the model using Bayesian inference.

The results showed a second wave of infections occurring after 2001 and 56,000 (95% Credible Interval 43,000–71,000) new infections in 2015, 37,000 (95% CrI 28,000–54,000) infections in men and 16,000 (95% CrI 10,000–23,000) in women. The estimated number of PLHIV by end-2015 was 838,000 (95% CrI 675,000–1,083,000), with 80% (95% CrI 62–98%) of those individuals reported to the Ministry of Health. Women were more likely to be diagnosed and reported than men; 86.8% of infected women had been reported compared with 75.7% of men. Likewise, ART initiation rates for women were higher than those for men.

The second wave contradicts previous estimates of HIV incidence trends in Brazil and there were persistent differences in the rates of accessing care between men and women. Nevertheless, the Brazilian HIV program has achieved high rates of detection and treatment, making considerable progress over the past ten years.

## Highlights

• We combined four surveillance networks of HIV/AIDS monitoring in Brazil.
• We used an age-structured deterministic model to infer HIV incidence.
• By 2001 a second wave of HIV infections was occurring in Brazil.
• There were persistent differences in linkage to care by sex.
• Of 838,000 people living with HIV by 2015, 80% were diagnosed and reported.

## 1 Introduction

The 90-90-90 goals established by the Joint United Nations Programme on HIV/AIDS (diagnose 90% of all people living with HIV [PLHIV], treat 90% of those diagnosed and achieve viral suppression in 90% of those treated by 2020) are key targets for ending the AIDS epidemic (Joint United Nations Programme on HIV/AIDS, 2014). Brazil’s ambitious and progressive approaches to fighting HIV/AIDS initially led to significant improvements in survival rates and falling incidence rates by guaranteeing free highly active antiretroviral therapy (HAART) under Federal Law since 1996 (Fonseca and Bastos, 2007; Marins et al., 2003; Ministério da Saúde Brasil, 1999). The early HIV epidemic in Brazil was driven by transmission within highly connected groups of men-who-have-sex-with-men (MSM) in the larger cities of the South-East. During the 1990s, the epidemic shifted towards the general population with a greater proportion of women becoming infected and spread into smaller cities in the interior of Brazil (Fonseca et al., 2002; Bastos and Szwarcwald, 2000; Fonseca et al., 2003; Szwarcwald et al., 2000). Intravenous drug users represent a very small proportion of PLHIV which has declined over time (Department of STI AIDS and Viral Hepatitis, 2015). Our current understanding of the epidemic suggests a stable (Department of STI AIDS and Viral Hepatitis, 2015) or slowly increasing national trend in HIV incidence (Szwarcwald et al., 2015; Wang et al., 2016; Joint United Nations Programme on HIV/AIDS (UNAIDS), 2017). Emerging evidence suggests a more worrying situation, with the number of new infections rising between 2010 and 2017 (AIDSinfo, 2019) and the spread of infection into more remote areas such as the Northern region along with increases in the numbers of reported cases in larger coastal cities in the North-East (Ministério da Saúde Brasil, 2016).

Back-calculation methods using B-splines to describe a flexible force of infection have been applied to routine case surveillance data across multiple settings to provide estimates of the size of the HIV epidemic and undiagnosed population (van Sighem et al., 2015; Vesga et al., 2014; Hogan and Salomon, 2012). These methods have shown promising results, even in concentrated epidemics with sparse data and the inclusion of data on CD4 cell counts allows trends in incidence to be distinguished from changes in diagnosis rates. Using a fixed force of infection may not have sufficient flexibility to model the HIV epidemic in Brazil, where changing risk behaviours and large-scale ART rollout may have caused rapid changes in individual risk.

Brazil has a wealth of surveillance data managed centrally by the Ministry of Health. Using these integrated health information systems, it is possible to track an individual’s progress through the health care system, from diagnosis, to CD4 cell count measurements, treatment and death. Here we describe a mathematical back-calculation model incorporating age and sex structure, developed to synthesise all available surveillance data in Brazil (case-reports, CD4 data, ART initiation, mortality reports and under-reporting of deaths) within a formal Bayesian framework to produce estimates of the HIV epidemic. We reproduce the complex multi-faceted surveillance systems in Brazil, incorporating the introduction of newer surveillance systems in 2001 and 2006 and more recent programmatic changes affecting reporting and linkage to produce estimates of incidence and number of PLHIV by age and sex, reporting rates and subsequently the size of the undiagnosed population.

## 2 Methods

### 2.1 Surveillance data

Detailed clinical and demographic data were extracted from four sources of anonymised surveillance data routinely collected during 1980–2016 by the Department of Sexually Transmitted Infections, AIDS and Viral Hepatitis (DIAHV) within the Ministry of Health, Brazil: notifiable diseases surveillance (SINAN, 1980–2016), two independent clinical monitoring systems (CD4 cell counts [SISCEL, 2001–2016] and ART dispensations [SICLOM, 2006–2016]) and mortality records (SIM, 1980–2015). Individual-level data on deaths due to AIDS-related causes are reported in SIM from 2000 onwards (aggregated annual values are available from 1980). To improve the incomplete reporting of AIDS cases via SINAN, data obtained via the SISCEL, SICLOM and SIM reporting systems were individually linked using a probabilistic algorithm developed by the DIAHV, duplicate records were matched, consolidated and then anonymized (Fonseca et al., 2010). National-level mortality records from SIM (pre-2000) and data on misclassification of AIDS deaths (1985–2009) supplemented the anonymised individual-level data (Fazito et al., 2012).

If CD4 cell count measurements at the time of reporting (defined as a CD4 cell count within six months of the first date that an individual appears in any of the surveillance databases) or at ART initiation were missing (CD4 cell count up to three months before starting ART), they were imputed by fitting probability distributions to available CD4 data by age, sex and calendar year and assigning a random draw from these (see Supplementary Information). We assumed that the distributions of CD4 counts for those with CD4 measurements were representative of those missing these data (Fig S1). All data were disaggregated by age in years and smoothed over time using non-parametric Loess regression to reduce the influence of artificial peaks in the data which occurred after the introduction of each new surveillance system.

### 2.2 Model structure

**Fig. 1:**
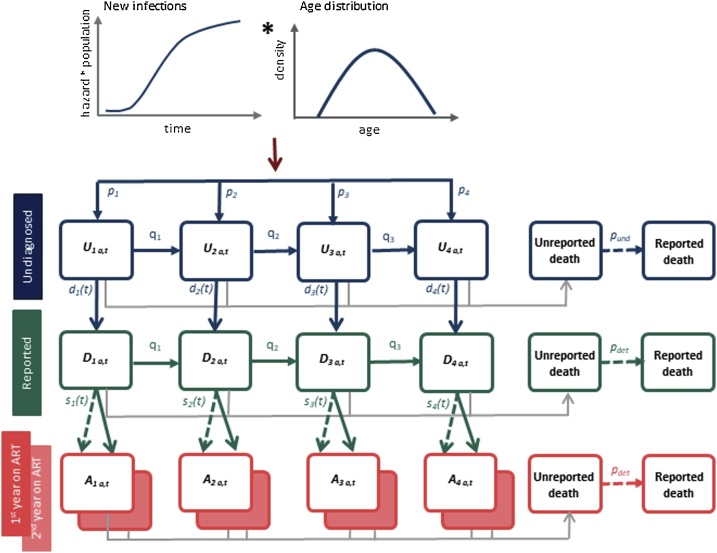
Simplified schematic representation of the age-structured compartmental model representing the movement of individuals between CD4 states at time *t* according to their current age in years *a*. The dashed lines moving from detected states to ART states refer to those initiating treatment at the time of diagnosis.

The natural history of HIV, along with the stages of reporting, ART initiation and death were characterised in a discrete-time age-structured deterministic model, with transitions between compartments described by partial differential equations (Fig. 1, Supplementary Table S1). Separate models were constructed for males and females, allowing all parameters to be estimated independently by sex.

Following infection, individuals were allocated into the appropriate age strata according to an age distribution curve before entering the first series of compartments (*U_i_* where *i* = 1, …, 4) characterised by CD4 cell count ranges (≥500, 350–499, 200–349, <200 cells/μL) according to the initial state probability (*p_i,a_* where *a* refers to the individual’s age). Individuals could then either progress, moving to states corresponding with lower CD4 cell counts according to the progression rates (*q_i_*), or become reported through one of the routine surveillance programmes (i.e. at initial HIV diagnosis, when CD4 counts were measured, when ART was started or when diagnosed with AIDS) at rate *d_i,t_*. The reporting rates were estimated separately for each CD4 state and assumed a piecewise linear function over time, with breakpoints at 1985, 2001 and 2010 corresponding with the start of the national clinical monitoring system and national scale-ups in surveillance networks in Brazil.

Upon reporting, individuals could then initiate ART and move into the compartments (*A_i_* where *i* = 1, …, 4), dependent on their CD4 count at ART initiation. Progression was not modelled explicitly once ART had started and mortality rates on treatment depended on the CD4 count at ART initiation, age and sex. We determined the proportion of cases by age-group (*r_i,a,t_*) first reported via their ART prescriptions and allowed this proportion to move directly to one of the ART states (dashed lines in Fig. 1), dependent on their CD4 count at reporting time (and simultaneous ART initiation). ART initiation rates (*s_i,t_*) of all others (1- *r_i,a,t_*) were estimated by calibration to the data. ART initiation rates were described by four piecewise linear functions over time, estimated separately for each CD4 count. ART initiation rates were constant during 1997–2000, then increased with gradients changing at 2010.

Mortality due to AIDS (μ*_i_*) could occur from every compartment along with background non-AIDS mortality (Mangal, 2017; United Nations Department of Economic and Social Affairs Population Division, 2017). With concentrated epidemics, such as in Brazil, mortality in the general population due to AIDS is low and so all-cause mortality rates were used as a proxy for background non-AIDS death rates. Mortality rates for the first and subsequent years on ART were estimated from the data using survival analysis (see Supplementary Information). The mean time from infection to reporting was estimated by summing the average time to entry into states *D_i_* (*i* = 1, … 4) via all possible pathways, weighted by the probability of following that pathway using the following equation adapted from van Sighem et al. (2015):

$$t_{rep}=\sum_{k=1}^{4}p_{k}\sum_{j=k}^{4}\frac{d_{j}(t)}{q_{j}+d_{j}\left(t\right)+\mu_{i}}\left(\prod_{s=k}^{j-1}\frac{q_{s}(t)}{q_{s}+d_{s}\left(t\right)+\mu_{s}}\right)\sum_{i=k}^{j}\frac{1}{q_{i}+d_{i}\left(t\right)+\mu_{i}}$$

We calculated mean time to reporting from 2001 onwards, i.e. following the launch of the national SISCEL system for monitoring CD4 counts, which we use as a reliable indicator of time since infection.

### 2.3 Incidence spline

Cubic B-splines represented the hazard of infection over time (van Sighem et al., 2015; Vesga et al., 2014; Brown et al., 2014; Bao, 2012). The knot vector *V* was a set of non-decreasing numbers where *v_0_* ≤ *v_1_* ≤ … ≤ *v_n_* and each knot position was optimised during the calibration process. The hazard curve was parameterised using ten basis functions over the time period 1965–2025 and the *n*-th B-spline basis function (*B_n,p_*(*v*)) with degree *p* = 3 can be defined using the Cox-de Boor recursion formula:

$$B_{n,0}\left(v\right)=\left\{\begin{array}{cc} 1 & if\;v_{n}\leq v<v_{n+1}\\ 0 & otherwise \end{array}\right)$$

$$B_{n,p}\left(v\right)=\frac{v-v_{n}}{v_{n+p}-v_{n}}N_{n,p-1}\left(v\right)+\frac{v_{n+p+1}-v}{v_{n+p+1}-v_{n+1}}N_{n+1,p-1}(v)$$

The first three basis function coefficients were fixed at zero, also setting the first derivatives of incidence to zero, to anchor the incidence at zero during the early epidemic phase and the remaining basis function coefficients were estimated during the model fitting. We calibrated three sets of models with differing numbers of internal knots (Ministério da Saúde Brasil, 1999; Fonseca et al., 2002; Bastos and Szwarcwald, 2000) and used the Bayesian Information Criterion (BIC) to select the most parsimonious model capable of representing the data.

### 2.4 Model calibration to surveillance data

The individual-level surveillance data used to calibrate the model were (i) reported cases of HIV/AIDS, (ii) CD4 cell counts, (iii) ART prescriptions dispensed and (iv) AIDS deaths. A joint likelihood described the probability of observing these data in a given month assuming an underlying Poisson distribution. Additional aggregated (national-level) data on numbers of AIDS deaths pre-2001 and misclassification rates of AIDS deaths (Fazito et al., 2012) were used to constrain the incidence curve under the assumption that these measures combined provided the total number of AIDS deaths that occurred. Model inputs and estimated model parameters along with their prior distributions are defined in the Supplementary Information (Tables S1 and S2). The calibration of the model to the smoothed data was conducted using parallel Markov chain Monte Carlo (MCMC) methods using diffuse uniform priors spanning a wide credible range (Supplementary Table S2). A multivariate Gaussian proposal distribution accounted for the correlations between parameters and a Robbins-Monro update step automatically tuned the scale parameter of the random-walk Metropolis-Hastings (MH) sampler (Garthwaite et al., 2016). The covariance matrix was estimated jointly with the scale parameter allowing convergence to be reached more efficiently. Fifteen MCMC chains each for the male and female models were initiated using different starting values from within a credible range and run for 500,000 iterations. An initial burn in of 50,000 iterations was discarded from each chain and one in ten iterations were subsampled from each chain to reduce autocorrelation. Chains were visually inspected for convergence and median parameter estimates along with 95% credible intervals (Cr I) were derived from the posterior distributions. All analyses were conducted in R, version 3.2.2.

### 2.5 Inferred incidence trends

We recreated the HIV incidence curve by randomly drawing 1000 parameter sets from the joint posterior distributions. Median incidence estimates, number of PLHIV, reporting rates, ART initiation rates and the size of the undiagnosed population were estimated for each parameter set and credible intervals were obtained using the 2.5th and 97.5th percentiles. An uncertainty analysis explored the impact of our assumptions on the natural history of HIV and the incidence spline function on the model outcomes (see Supplementary information). Outputs are presented up to 2015, as this is the last full year for which we have data. Estimates of the number of new infections and PLHIV are rounded to the nearest thousand.

### 2.6 Role of the funding source

The funding bodies had no role in the study design, collection, analysis, interpretation of data or in the writing of the report and the decision to submit the paper for publication.

### 2.7 Ethical consent

Written informed consent from individuals and ethical approval was not required as this was a retrospective analysis of fully anonymised surveillance data collected during routine HIV case monitoring.

## 3 Results

### 3.1 Surveillance data

**Fig. 2:**
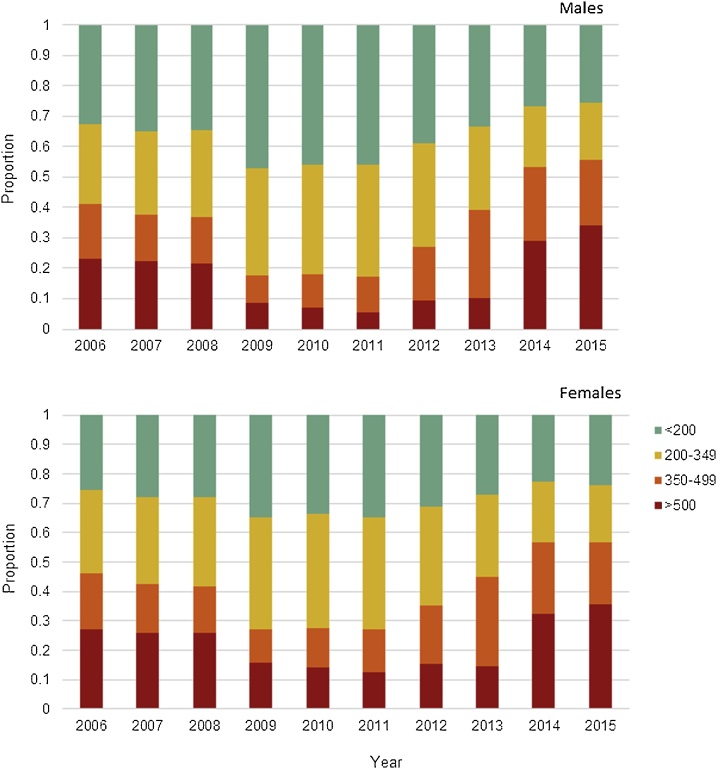
The proportion of people living with HIV initiating ART in each of the four CD4 states (>500, 350–499, 200–349 and < 200 cells/μl) for men (upper panel) and women (lower panel) during 2006–2015. CD4 cell counts are reported via SISCEL and we do not include augmented data in this figure.

A total of 1,077,295 individuals with HIV were reported between January 1980 and August 2016, with 298,159 reported deaths. CD4 cell counts were available for 662,720 (61.5%) individuals, and 654,676 (60.8%) had at least one ART prescription recorded. The proportion initiating ART by CD4 cell count over time is shown in Fig. 2. The mean age at the time of reporting ranged from 33.1 years (SD 9.6) in 1990 to 35.6 years (SD 12.1) in 2015, with the majority of cases (87.8%) aged between 15–49 years. The male to female ratio of reported cases changed from 5.7:1 in 1990 to 1.4:1 in 2002 and 2.3:1 in 2015.

### 3.2 Estimated trajectory of the epidemic

**Fig. 3:**
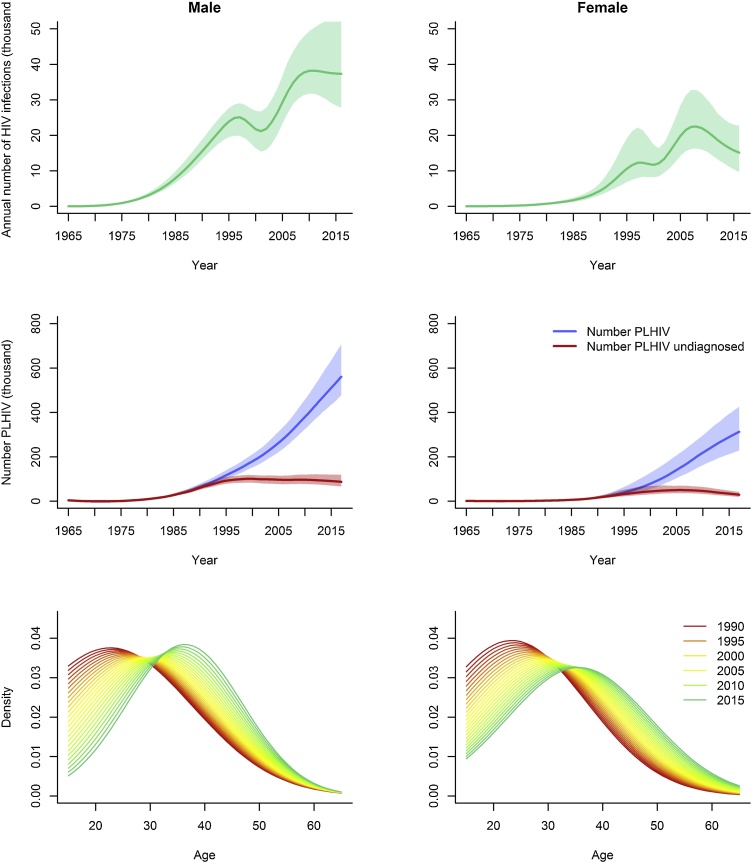
The HIV epidemic in Brazil 1980–2015 in men (left panels) and women (right panels). Top row: Annual number of new HIV infections in adults aged 15 years and above. Middle row: Total number of adults living with HIV/AIDS (blue) and the number of undiagnosed infections (red). Both sets of figures show results using the median value from the posterior distributions (solid lines) with 95% credible intervals (shaded areas). Bottom row: Age distribution of new HIV infections 1990–2015 (For interpretation of the references to colour in this figure legend, the reader is referred to the web version of this article).

The epidemic peaked first in 1997 with an incidence rate of 0.348 / 1000 person-years (95% credible interval 0.259 – 0.477 / 1000 py), settling to 0.284 / 1000 py (0.204 – 0.370 / 1000 py) by 2001. The epidemic curves show a “second wave” of infections occurring in both men and women from 2001 onwards (Fig. 3). The double peak was observed in all 1000 incidence curves generated by sampling from the joint posterior distributions. Between 2001 and 2009, incidence rates increased by 56.4% and 59.8% in men and women respectively to 0.574 and 0.326 / 1000 person-years.

Over time, there has been a shift in the age distribution of new HIV cases, with the peak age at infection increasing from 23 years in 1990 to 36 years in 2015 for men and women and a higher number of infections occurring in older age groups (Fig. 3).

In 2015, 56,000 (95% Cr I 43,000–71,000) new infections occurred, 37,000 (95% Cr I 28,000–54,000) infections in men and 16,000 (95% Cr I 10,000–23,000) in women. The number of PLHIV by end-2015 was 838,000 (95% Cr I 675,000–1,083,000).

### 3.3 Estimates of reporting rates, treatment initiation and mortality reporting

Prior to 1997, only those with AIDS diagnoses are reported and consequently, the highest reporting rates are found in those with low CD4 counts of <200 cells/μL (1.11yr^−1^, 95% Cr I 0.89–1.51 for men in 1996 and 1.06yr^−1^, 95% Cr I 0.89–1.38 for women). The reporting rates for those with the highest CD4 counts (CD4 ≥ 500 cells/μL) have tripled over the past ten years, due to the launch of the ART dispensation reporting system (from 2006 onwards) and the expansion of the clinical reporting networks (Fig S2). The mean time overall from infection to diagnosis decreased over time, from 5.05 and 5.16 years for men and women respectively in 2001 to 1.33 and 0.76 years in 2015 (Fig S3).

**Fig. 4:**
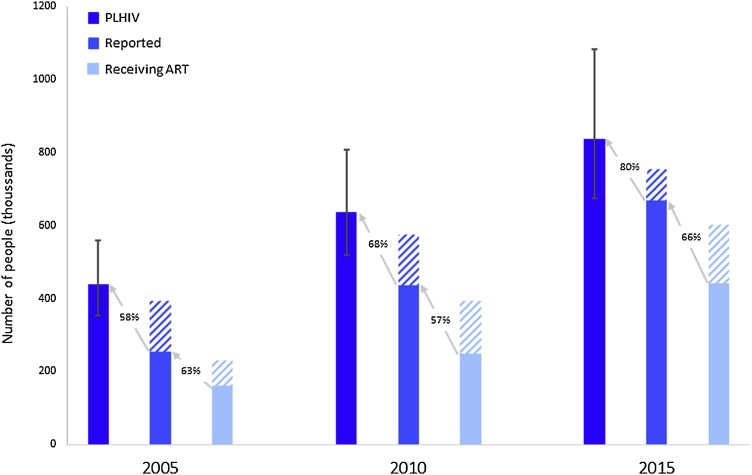
Number of people aged over 15 years living with HIV (PLHIV) in Brazil, number reported and number on antiretroviral treatment (solid bars) and the gaps to reaching the first and second of the UNAIDS 90-90-90 targets (shaded areas). Estimates of the number of PLHIV are derived from the model outputs for the end of 2005, 2010 and 2015 and show the associated uncertainty. Numbers of reported and treated individuals up to the end of each year are published by the Ministry of Health, Brazil (Ministério da Saúde Brasil, 2016; Ministério da Saúde, Brasil, 2017a). The percentage values between the bars represent the percentage of PLHIV who have been reported and the percentage of those reported who are on treatment for each year.

By 2015, 80% (95% Cr I 62–99%) of the estimated 838,000 PLHIV in Brazil were aware of their status and reported to the DIAHV, compared with 58% (46–72%) and 68% (54–84%) in 2005 and 2010 respectively (Fig. 4). Women were more likely to be reported than men; by the end of 2015, 86.8% of women living with HIV had been documented by one of the surveillance networks compared with 75.7% of men.

Treatment initiation rates have increased over time for all CD4 states, with the highest rates estimated for those with CD4 cell counts 350–499 cells/μL (0.13, 95% Cr I 0.10 – 0.18 for men and 0.20, 95% Cr I 0.14 – 0.34 for women in 2015, Fig S4). At all CD4 counts, estimated treatment initiation rates were higher for women than men and these differences increased over time.

The proportion of deaths in known HIV/AIDS cases that were reported via the Mortality Information System remained almost constant over time, 0.91 (95% Cr I 0.84 – 0.99) for men in 2001 to 0.92 (95% Cr I 0.85 – 0.99) in 2015. Similarly, for women, the proportion of deaths reported for known cases remained constant at 0.97 (95% Cr I 0.86–1.00) in 2001 and 0.98 (95% Cr I 0.88–1.00) in 2015. Reporting of AIDS-related deaths in unreported cases increased slightly over time, from 0.56 (95% Cr I 0.53-0.63) and 0.60 (95% Cr I 0.53 – 0.66) in men and women respectively in 2006, to 0.62 (95% Cr I 0.59 – 0.70) and 0.66 (95% Cr I 0.58 – 0.73) in 2015, meaning over 60% of cases who are unreported are subsequently identified post-mortem.

### 3.4 Model calibration

**Fig. 5:**
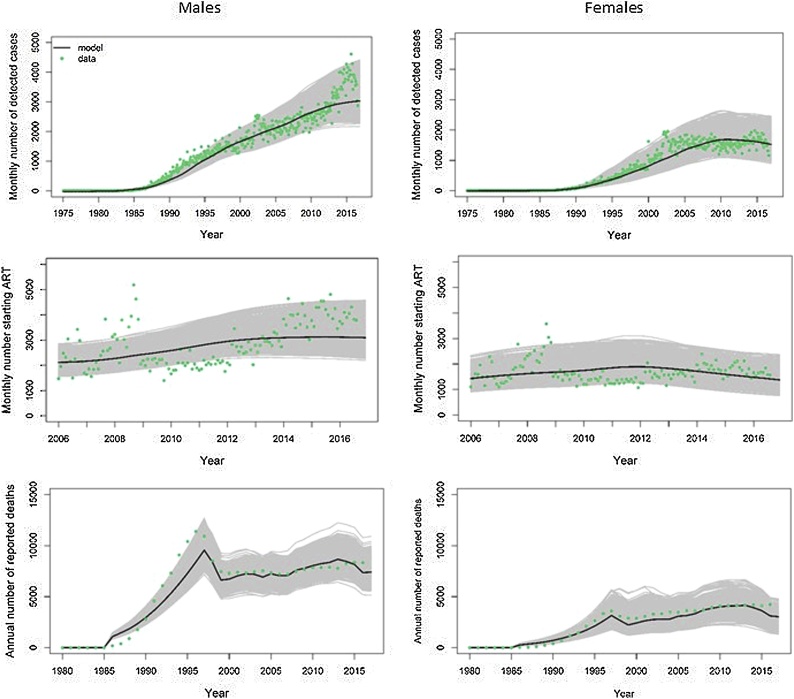
Calibration of the model (black / grey lines) to observed data (green points) on the numbers of newly detected cases by month (first row), number of first ART prescriptions by month (second row) and annual numbers of reported deaths (third row). The median values from the posterior distributions are shown in black and the grey lines represent the estimated uncertainty (For interpretation of the references to colour in this figure legend, the reader is referred to the web version of this article).

In general, there is good agreement between the model and the data (Fig. 5). We did not explicitly fit the model to the peaks in reported cases observed in 2001–2002 or the peaks in ART cases observed in 2007–2008 as they are due to the implementation of the laboratory systems database (SISCEL in 2001) and the ART dispensation system (SICLOM in 2006) and therefore represent temporary increases in reporting rates. The estimates of male deaths before 1997 are slightly lower than the observed values, although the majority of the observed data points lie within the uncertainty bounds of the model. One potential explanation for this is that we are under-estimating the mortality rates of HIV-infected males during the early period of the epidemic or possibly under-estimating the number of men infected during this period.

### 3.5 Uncertainty analysis

An additional sixteen models were developed using alternative parameter sets which included different initial state probabilities following seroconversion, CD4 progression rates and mortality rates, grouping parameters into high versus low initial CD4 counts at seroconversion, fast versus slow progression and high versus low mortality rates. The incidence curves produced were qualitatively similar across all parameter sets, producing a double-peaked epidemic curve with small shifts in the size and timing of the peaks. The effects of changing the natural history assumptions on the incidence trends in women were less pronounced than for men. The ranges in median incidence in 2015 estimated from these alternative analyses were 32,000–52,000 for men and 13,000–20,000 for women. More details are presented in the Supplementary Information.

## 4 Discussion

The findings suggest there has been a second wave of infections since 2001 which has been stabilising since 2010, contradicting previous estimates and official positions on the HIV incidence trends in Brazil (AIDSinfo, 2019; IHME, 2017; Szwarcwald et al., 2016a). This second peak has been speculated upon, given the disproportionately high incidence of infection recently reported in men, but this is the first time compelling evidence has been presented for it (Szwarcwald et al., 2016b; de Castro et al., 2010). Given these findings, it is arguably not surprising to see further evidence of a resurgence of the epidemic in men, however the second peak in incidence in women has not previously been documented. Other settings have experienced similar rises in incidence amongst MSM, indicating that our findings here may provide insights into other regions (Beyrer et al., 2013; Van Sighem et al., 2012).

Nevertheless, Brazil has achieved high reporting and treatment rates; by 2015 80% (95% Cr I 62–99) of PLHIV were reported and the Ministry of Health reported 442,000 PLHIV were accessing ART (i.e. 66% of reported cases were treated) (Ministério da Saúde, Brasil, 2017a). Though not yet achieving the UNAIDS targets for 2020, the Brazilian HIV/AIDS programme compares favourably to the performance of most other national programmes (globally 70% [51–84%] of PLHIV know their status and 77% [57–89%] of those are on treatment) (Joint United Nations Programme on HIV/AIDS (UNAIDS), 2017). Our estimates of the proportion of PLHIV reported are in line with others; Levi et al reported 80% diagnosed and 60% of those on ART in 2013 (Levi et al., 2016). However, both Levi et al and the Ministry of Health report low viral suppression levels (35%–50%) indicating potentially poor ART adherence (Ministério da Saúde, Brasil, 2017a).

Such an increase in incidence in recent years has gone undetected and unreported until now. The UNAIDS-supported Spectrum tool generated estimates of 48,000 (95% CI 34,000–62,000) new infections in 2015, with similar numbers of PLHIV to those described here (800,000 95% CI 580,000–1,000,000) (AIDSinfo, 2019; Stover et al., 2017). The Global Burden of Disease Study (Wang et al., 2016) assumes a slowly increasing epidemic trend similar to that of UNAIDS, also with a lower number of new infections (33,790, 95% CI 30,200–37,520 in 2015) but yielding an estimate of 558,840 (95% CI 454,380–687,400) PLHIV, lower than the official number of PLHIV linked to care (Pascom et al., 2017). Our estimates of incidence between 2004 and 2010 are higher than those published by Szwarcwald et al. which are generated using a single surveillance database, however estimates do overlap in more recent years (Szwarcwald et al., 2016a). Using a phenomenological approach allows us to be agnostic about the incidence trends and the spline is not constrained with prior beliefs about the changing impact of the AIDS programme. The sensitivity analysis shows that the findings are robust to alternative assumptions on the functional form of the spline.

The flexible reporting rates were incorporated to allow for newer surveillance systems being implemented and for initiatives aimed at promoting testing, which may lead to a greater proportion of PLHIV being diagnosed (Department of STI AIDS and Viral Hepatitis, 2015). For this reason we see large increases in reporting rates in the highest CD4 states (≥500 cells/μL), corresponding with increasingly early diagnoses. This is particularly pronounced for women where estimated reporting times fell to approximately four months by 2015 for the 59% who seroconvert into the highest CD4 state. The fall in mean time from infection to reporting, from approximately five years at the inception of the Laboratory Tests Control System (SISCEL) in 2001 to 1.33 and 0.76 years for men and women respectively by the end of 2015, was consistent with earlier findings showing that by 2013, 35.2% of men and 45.8% of women were diagnosed in the same year as they were infected (Szwarcwald et al., 2016a). By 2015 we see a peak age at infection similar to the mean age at the time of reporting because of this short reporting delay.

The recent HIV Biological Behavioural Surveillance Surveys showed changes in risk-taking behaviour in MSM over the last ten years which varied by age, for example, the proportion of MSM aged 25 years and older with more than six sexual partners has increased by 45% since 2006 compared with a decrease of 2% in <25 year olds (Guimarães et al., 2018). In contrast, the proportion of young MSM reporting unprotected receptive anal intercourse has increased by 24% compared with an 18% decrease in older MSM. Here we find an increased risk of HIV in older individuals in recent years, consistent with reports of increases in multiple partnerships, however the functional form of the age distribution curve did not allow for a bimodal distribution in risk and so it’s possible that a more flexible function may show an increase in risk for both younger and older age groups.

Interestingly, the highest ART initiation rates are in those with CD4 counts between 350–499 cells/μL, suggesting that those people who are diagnosed early are then starting treatment early. The estimated reporting and ART initiation rates suggest two distinct populations, one group who are detected soon after infection with high CD4 counts and then treated early, possibly due to the expansion of rapid HIV testing and outreach programmes or the presence of an illness associated with HIV, and a second group who are not accessing HIV care until the late stages of infection (with CD4 counts <200 cells/μL). These inequalities in care have previously been documented, with some high-risk groups facing significant barriers to testing (de Barros et al., 2017) and treatment (Teixeira et al., 2014; Ministério da Saúde, Brasil, 2017b; Grangeiro et al., 2011).

Disentangling changes in incidence from changes in reporting rates can be problematic and so we allow for biases in reporting and simplify testing and linkage where we do not have much information. The flexible spline function describing the hazard of infection allows rapid changes and it is possible that using a more rigid function would preclude the detection of a double peak in incidence.

This analysis reveals a strong and persistent differential in the rates of accessing care between men and women. By 2015, 86.8% of women living with HIV had been reported, compared with 75.7% of men, likely due in part to the high HIV testing coverage during antenatal care (Botelho CAO, 2008; Botelho et al., 2008). We do not consider treatment adherence but model treatment initiation only. Viral suppression is not modelled here, but has important implications for transmission risk and is required for generating projections of the future burden of HIV.

Our findings rely on using back-calculations to estimate incidence using CD4 cell counts at the time of reporting. This method has the limitation of increasing uncertainty in more recent years, as newly reported cases provide information only on past incidence. Incidence assays can identify recently acquired infections and could be incorporated to improve estimations, but as yet, have been trialled in only a few small studies in limited regions in Brazil (Szwarcwald et al., 2016b; de Castro et al., 2010; Grinberg et al., 2015). Information on the CD4 progression rates and mortality without ART are not available for Brazil and we therefore acquired data from other sources, conducting an uncertainty analysis to assess the robustness of our results under a range of assumptions. CD4 cell counts were available for 75.4% of individuals after 2001 and we assume that those individuals are representative of the population. Individuals accessing care via private clinics would only be detected at the time of an AIDS diagnosis or by receipt of ART. Consequently we may be missing clinical monitoring data that could affect our assumptions on reporting rates.

Many important parameters needed here are estimated directly from the data, e.g. ART survival rates and CD4 distributions are derived from evaluations of the national-level surveillance programmes. One major strength of this approach is the use of case-reporting data which is routinely collected by the HIV/AIDS programmes in many countries to estimate incidence. There are no assumptions needed on the size or behaviour of susceptible populations which is difficult to obtain for many settings. Furthermore, we do not rely on adjustments of prevalence estimates from ante-natal clinic data or surveys.

This analysis shows that a second wave of infections occurred in Brazil, possibly due to higher risk behaviour resulting from a lower perceived risk of HIV, poor adherence to ART leading to rebounding viral loads or a sizeable undiagnosed population unaware of their infection status. To date, 31 municipalities in Brazil have signed the 2014 Paris Declaration, pledging to end AIDS as a public health threat by 2030 (Joint United Nations Programme on HIV/AIDS (UNAIDS), 2017), however, there is an urgent need to understand where and among whom new infections are occurring.

## Author contributions

Dr Mangal and Dr Pascom contributed equally to the manuscript. Specific contributions are: Literature search TDM, ARPP; Figures TDM; Study design TDM, TBH, ARPP, JFV; Data collection ARPP, MVM, ASB; Data analysis TDM, TBH, ARPP, JFV; Data interpretation TDM, TBH, ARPP, MVM, ASB, JFV; Writing: TDM, TBH, MVM, ARPP.

## Sources of funding

Title of Grant: The HIV Modelling Consortium.

Grant ID: OPP1084364.

Title of Grant: Multi-Country HIV Prevention Impact Modeling Study.

Contract Number: 7161402.

## References

[bib0005] AIDSinfo [Internet]. UNAIDS. 2017 [cited August 2017]. Available from: http://aidsinfo.unaids.org/.

[bib0010] Bao L. (2012). A new infectious disease model for estimating and projecting HIV/AIDS epidemics. Sex. Transm. Infect..

[bib0015] Beyrer C., Sullivan P., Sanchez J., Baral S.D., Collins C., Wirtz A.L. (2013). The increase in global HIV epidemics in MSM. AIDS.

[bib0020] Botelho C.A.O., Tomaz C.A.B., Cunha R.V., Botelho M.A.O., Botelho L.O., Assis D.M. (2008). Prevalência dos agravos triados no programa de proteção à gestante do Estado do Mato Grosso do Sul de 2004 a 2007. Rev. Patol. Trop..

[bib0025] Botelho CAO (2008). Prevalência dos agravos triados no programa de proteção à gestante do estado de Mato Grosso do Sul de 2004 a 2007.

[bib0030] Brown T., Bao L., Eaton J.W., Hogan D.R., Mahy M., Marsh K. (2014). Improvements in prevalence trend fitting and incidence estimation in EPP 2013. AIDS.

[bib0035] de Barros C., Sabidó M., de Miranda Lobo T., Pascom R., Pasini E. (2017). Community-based rapid HIV testing in Brazil for vulnerable populations: whom are we reaching?. J. AIDS Clin. Res..

[bib0040] de Castro C.A.V., Grinsztejn B., Veloso V.G., Bastos F.I., Pilotto J.H., Morgado M.G. (2010). Prevalence, estimated HIV-1 incidence and viral diversity among people seeking voluntary counseling and testing services in Rio de Janeiro, Brazil. BMC Infect. Dis..

[bib0045] Department of STI AIDS and Viral Hepatitis (2015). Global AIDS Response Progress Reporting Narrative Report – Brazil.

[bib0050] Fazito E., Cuchi P., Ma Fat D., Ghys P.D., Pereira M.G., Vasconcelos A.M.N. (2012). Identifying and quantifying misclassified and under-reported AIDS deaths in Brazil: a retrospective analysis from 1985 to 2009. Sex. Transm. Infect..

[bib0055] Bastos F.I.P.M., Szwarcwald C.L. (2000). AIDS e pauperização: principais conceitos e evidências empíricas. Cad Saude Publica.

[bib0060] Fonseca M.G.P., Bastos F.I. (2007). Twenty-five years of the AIDS epidemic in Brazil: principal epidemiological findings, 1980–2005. Cad Saude Publica.

[bib0065] Fonseca M.G.P., Szwarcwald C.L., Bastos F.I. (2002). Análise sociodemográfica da epidemia de Aids no Brasil, 1989–1997. Rev. Saude Publica.

[bib0070] Fonseca M.G.P., Travassos C., Bastos F.I., Silva N.V., Szwarcwald C.L. (2003). Social distribution of AIDS in Brazil, according to labor market participation, occupation and socioeconomic status of cases from 1987 to 1998. Cad Saude Publica.

[bib0075] Fonseca M.G.P., Coeli C.M., Lucena F.F.A., Veloso V.G., Carvalho M.S. (2010). Accuracy of a probabilistic record linkage strategy applied to identify deaths among cases reported to the Brazilian AIDS surveillance database. Cad Saude Publica.

[bib0080] Garthwaite P.H., Fan Y., Sisson S.A. (2016). Adaptive optimal scaling of Metropolis–Hastings algorithms using the Robbins–Monro process. Commun. Stat. Theory Methods.

[bib0085] Grangeiro A., Escuder M.M., Menezes P.R., Alencar R., Ayres de Castilho E. (2011). Late entry into HIV care: estimated impact on AIDS mortality rates in Brazil, 2003–2006. PLoS One.

[bib0090] Grinberg G., Giron L.B., Knoll R.K., Galinskas J., Camargo M., Arif M.S. (2015). High prevalence and incidence of HIV-1 in a counseling and testing center in the city of Itajaí, Brazil. Braz. J. Infect. Dis..

[bib0095] Guimarães M.D.C., Kendall C., Magno L., Rocha G.M., Knauth D.R., Leal A.F. (2018). Comparing HIV risk-related behaviors between 2 RDS national samples of MSM in Brazil, 2009 and 2016. Medicine (Baltimore).

[bib0100] Hogan D.R., Salomon J.A. (2012). Spline-based modelling of trends in the force of HIV infection, with application to the UNAIDS Estimation and Projection Package. Sex. Transm. Infect..

[bib0105] Joint United Nations Programme on HIV/AIDS (2014). 90-90-90: An Ambitious Treatment Target to Help End the AIDS Epidemic.

[bib0110] Joint United Nations Programme on HIV/AIDS (UNAIDS) (2017). Ending AIDS. Progress Towards the 90-90-90 Targets.

[bib0115] Levi J., Raymond A., Pozniak A., Vernazza P., Kohler P., Hill A. (2016). Can the UNAIDS 90-90-90 target be achieved? A systematic analysis of national HIV treatment cascades. BMJ Glob. Health.

[bib0120] Mangal T.D. (2017). Joint estimation of CD4+ cell progression and survival in untreated individuals with HIV-1 infection. AIDS (Lond., Engl.).

[bib0125] Marins J.R.P., Jamal L.F., Chen S.Y., Barros M.B., Hudes E.S., Barbosa A.A. (2003). Dramatic improvement in survival among adult Brazilian AIDS patients. AIDS.

[bib0130] IHME (2017). Millenium Development Goals (MDGs) Visualization | Viz Hub [Internet]. https://vizhub.healthdata.org/mdg/.

[bib0135] Ministério da Saúde Brasil (1999). POLÍTICA NACIONAL DE DST/AIDS. PRINCÍPIOS, DIRETRIZES E ESTRATÉGIAS Brasilia.

[bib0140] Ministério da Saúde Brasil (2016). Boletim Epidemiológico – Aids e DST.

[bib0145] Ministério da Saúde, Brasil (2017). Relatório de Monitoramento Clínico do HIV.

[bib0150] Ministério da Saúde, Brasil (2017). Boletim Epidemiológico HIV AIDS 2017.

[bib0155] Pascom A.R., Barros C.H.D., Lima V.D., Freitas M.A., Montaner J.S.G., Meireles M. (2017). The HIV care continuum in Brazil, from 2009 to 2015 [abstract]. 9th IAS Conference on HIV Science.

[bib0160] Stover J., Brown T., Puckett R., Peerapatanapokin W. (2017). Updates to the Spectrum/Estimations and Projections Package model for estimating trends and current values for key HIV indicators. AIDS.

[bib0165] Szwarcwald C.L., Bastos F.I.P.M., Esteves M.A.P., Andrade C.L.T. (2000). A disseminação da epidemia da AIDS no Brasil, no período de 1987-1996: uma análise espacial. Cad Saude Publica.

[bib0170] Szwarcwald C., Pascom A., de Souza Júnior P. (2015). Estimation of the HIV incidence and of the number of people living with HIV/AIDS in Brazil, 2012. J. AIDS Clin. Res..

[bib0175] Szwarcwald C., de Souza Júnior P., Pascom A., Ferreira O. (2016). Results from a method for estimating HIV incidence based on the first Cd4 count among treatment-naïve cases: Brazil, 2004–2013. J. AIDS Clin. Res..

[bib0180] Szwarcwald C.L., Júnior F., da Costa O., Brito A.M., Luhm K.R., Ribeiro C.E.L. (2016). Estimation of HIV incidence in two Brazilian municipalities, 2013. Rev. Saude Publica.

[bib0185] Teixeira T.R.A., Gracie G., Malta M.S., Bastos F.I. (2014). Social geography of AIDS in Brazil: identifying patterns of regional inequalities. Cad Saude Publica.

[bib0190] United Nations Department of Economic and Social Affairs Population Division (2017). World Population Prospects: The 2017 Revision, Key Findings and Advance Tables.

[bib0195] van Sighem A., Nakagawa F., De Angelis D., Quinten C., Bezemer D., de Coul E.O. (2015). Estimating HIV incidence, time to diagnosis, and the undiagnosed HIV epidemic using routine surveillance data. Epidemiology (Cambridge, Mass.).

[bib0200] Van Sighem A., Vidondo B., Glass T.R., Bucher H.C., Vernazza P., Gebhardt M. (2012). Resurgence of HIV infection among men who have sex with men in Switzerland: mathematical modelling study. PLoS One.

[bib0205] Vesga J.F., Cori A., van Sighem A., Hallett T.B. (2014). Estimating HIV incidence from case-report data: method and an application in Colombia. AIDS.

[bib0210] Wang H., Wolock T.M., Carter A., Nguyen G., Kyu H.H., Gakidou E. (2016). Estimates of global, regional, and national incidence, prevalence, and mortality of HIV, 1980–2015: the Global Burden of Disease Study 2015. Lancet HIV.

