# Supplementary material for: Estimating HIV incidence from surveillance data indicates a second wave of infections in Brazil
Source: Epidemics. 2019 Jun;27:77–85. doi: 10.1016/j.epidem.2019.02.002 (PMC6543066; doi:10.1016/j.epidem.2019.02.002)
Supplement: Supplementary file 1 [file mmc1.docx]

**SUPPLEMENTARY INFORMATION**

**Data**

Four sources of individually-linked surveillance data routinely collected during 1980-2016 by the Department of Sexually Transmitted Infections, AIDS and Viral Hepatitis (DIAHV) within the Ministry of Health, Brazil were used: notifiable diseases surveillance reports for HIV/AIDS, two independent clinical monitoring systems (CD4 cell counts and ART dispensations) and mortality records. Notification of AIDS cases (using either the CDC classification or the Rio de Janeiro/Caracas criteria) has been compulsory since 1980 and is recorded using the Notifiable Diseases Information System database (SINAN). More recently, HIV diagnoses have also been reported via this network, becoming compulsory in 2014. CD4 cell counts are recorded for those attending public health clinics from 2001 onwards using the Laboratory Tests Control System database (SISCEL). ART dispensations are recorded via the Medication Logistics Control System database (SICLOM) which documents all ART dispensations (both in the public and private health care sectors) issued in Brazil from 2006 onwards. Deaths due to HIV/AIDS were reported using the Mortality Information System database which uses the International Classification of Diseases, Tenth Revision (ICD-10, codes B20-24). All data cleaning and linkage was performed by the Surveillance Unit in the DIAHV using names, sex, mother’s names, dates of birth and areas of residence.[1] Probabilistic record linkage used these identifying variables to link records from different health information systems, verifying under-reporting and investigating incomplete entries. Each entry was issued a unique code and then stripped of all identifying information before being analysed.

Any entries with duplicated identifying codes were separated from the database and one entry from each duplicated set was randomly chosen for inclusion in the final database. We discovered 16,220 (1.5%) entries with duplicated identifying codes and randomly selected 7,856 unique entries from these duplicated sets. We also excluded entries with missing data on date of birth (used to derive age at detection, age at ART initiation and age at death), sex and date of detection, resulting in a total of 1,077,295 entries in the database.

Age- and sex-specific HIV/AIDS mortality rates pre-ART are fixed for each CD4 state and are derived from seroconverter data in Africa. There may be some differences in both progression and mortality rates between Africa and Brazil, but in the absence of data on these rates from South America, we use those from Africa, given that the all-cause mortality rates in Brazil lie closer to the rates found in Africa than those in Europe (the other region for which sufficient data are available to form robust estimates of progression patterns).

**Data augmentation**

We imputed CD4 cell counts for those missing data at two key points during the disease process, namely the time of reporting and time of first ART prescription. We fitted probability distributions to CD4 cell counts recorded within 6 months of the reporting date by SINAN, SISCEL and SICLOM. Separate distributions were fitted by year of reporting for each surveillance system and additionally by age-group, sex and calendar year. We tested multiple distributions (including gamma, exponential, lognormal and Weibull) for each subgroup and found that a gamma distribution satisfactorily describes the distribution of CD4 cell counts for each group. CD4 cell counts for individuals with missing data were randomly drawn from the fitted distributions given the means of reporting, their age and sex, and year of reporting. Additionally, we fitted probability distributions to CD4 cell counts recorded in the three months prior to ART initiation for those with ART records and used these fitted distributions to infer CD4 cell counts for those without this data. These methods can reproduce the same trends in CD4 cell count distributions at the population-level, but it’s possible that a machine-learning approach may yield more accurate predictions at the individual-level.

**Model equations**

The model describes the infected population using a series of partial differential equations, with *t* and *a* representing time and age respectively. A spline function (detailed in the main text) describes the hazard of infection and is multiplied by the susceptible population to give the number of newly infected individuals. The newly infected population (*I*) is allocated to the relevant age strata according to the distribution detailed below (section Age Distribution) and is then divided into the four undetected compartments (U) according to the initial state probabilities (*p_i_*). Individuals move through unreported states, being reported at time-dependent rate *d_i_*. Progression rates (*q_i_*) are identical between unreported and reported states (D). Once reported, a proportion of people can start ART (A) either immediately (proportion *r_i_* derived from the surveillance data which is stratified by age, sex, CD4 count at ART initiation and calendar year) or at a rate *s_i_* estimated by fitting to the data. After one year on ART, mortality rates change as detailed in Table S6. Mortality due to HIV/AIDS (*μ_i_*) is included from every compartment and background non-AIDS mortality can occur at a rate determined by age, sex and year.[2] A summary of the natural history parameters is presented in Table S1. Parameters informing the spline function, rates of reporting, rates of ART initiation, proportion of deaths reported and the age distribution of new infections are calibrated to the data, resulting in 46 parameters estimated for each model (male and female, Table S2).

Undetected state 1 (CD4 ≥ 500)

$\frac{\partial U_{1}\left( a,t \right)}{\partial a}+\frac{\partial U_{1}\left( a,t \right)}{\partial t}=p_{1}I\left( t \right)-d_{1}U_{1}\left( a,t \right)-q_{1}U_{1}\left( a,t \right)-\mu_{1}U_{1}\left( a,t \right)$

Undetected states 2,3,4

$\frac{\partial U_{i}\left( a,t \right)}{\partial a}+\frac{\partial U_{i}\left( a,t \right)}{\partial t}=p_{i}I\left( t \right)+q_{i-1}U_{i-1}\left( a,t \right)-d_{i}U_{i}\left( a,t \right)-q_{i}U_{i}\left( a,t \right)-\mu_{i}U_{i}\left( a,t \right) \left( i=2,3,4 \right)$

Reported state 1 (CD4 ≥ 500)

$\frac{\partial D_{1}\left( a,t \right)}{\partial a}+\frac{\partial D_{1}\left( a,t \right)}{\partial t}=d_{1}D_{1}\left( a,t \right)-q_{1}D_{1}\left( a,t \right)-r_{1}D_{1}\left( a,t \right)-\left( {1-r}_{1} \right){s_{1}D}_{1}\left( a,t \right)-\mu_{1}D_{1}\left( a,t \right)$

Reported states 2,3,4

$\frac{\partial D_{i}\left( a,t \right)}{\partial a}+\frac{\partial D_{i}\left( a,t \right)}{\partial t}=d_{i}D_{i}\left( a,t \right)+q_{i-1}D_{i-1}\left( a,t \right)-q_{i}D_{i}\left( a,t \right)-r_{i}D_{i}\left( a,t \right)-\left( {1-r}_{i} \right){s_{i}D}_{i}\left( a,t \right)-\mu_{i}D_{i}\left( a,t \right)$

ART states 1-4

$\frac{\partial A_{i}\left( a,t \right)}{\partial a}+\frac{\partial A_{i}\left( a,t \right)}{\partial t}=r_{i}D_{i}\left( a,t \right)-\left( {1-r}_{i} \right){s_{i}D}_{i}\left( a,t \right)-\mu_{i}A_{i}\left( a,t \right)$

**Age distribution**

The age distribution of new HIV cases was characterised using a simple three-parameter function which represents the relative hazard of infection by age, *g(x)* using parameters *α*, *β* and *γ*, describing the level, spread and skew of the curve respectively between the upper (*U*) and lower (*L*) age bounds:

$$g\left( x \right)=\left\{ \begin{matrix} \begin{matrix} \alpha\cdot exp\left( \frac{{-\left( x-\gamma U \right)}^{2}}{\beta\left( \frac{U}{2} \right)^{2}} \right) & , for L \leq x <U \\ 0 & , otherwise \end{matrix} \\ \end{matrix} \right.$$

Parameters *α*, *β* and *γ* are estimated along with a linear scaling factor for each parameter (*τ*, *υ*, *φ*) describing the change in level, spread and skew of the age distribution curve over time.

**Detection rates**

Data on HIV testing patterns and delays between testing and linkage to care in Brazil are sparse, therefore the reporting rates that we estimate here incorporate both the unknown testing rates along with the time delay before the CD4 count is taken, the AIDS diagnosis or the ART initiation. The reporting dates therefore may occur some time after the initial diagnosis, which is often not reported. The probability of being reported whilst in state *U_i_*(*t*) and moving to state *D_i_*(*t*) is *d_i_*(*t*)/*q_i_* + *d_i_*(*t*), where the progression rate *q_i_* remains fixed over time. We fixed the reporting rates to zero for CD4 states *U_i_* where *i*=1,2 (i.e. CD4 ≥ 350 cells/ μl) for the years 1980-1985, as cases could only be identified by the onset of AIDS during this time. Reporting rates during this period for *U_3_* and *U_4_* (CD4 200-349 and CD4 < 200 cells/ μl) were fixed so that mean time from infection to reporting was 4.19 and 7.93 years respectively.[3]

**Mortality data**

The Mortality Information System data is available from 1980 onwards and is subject to misclassification and under-reporting. To correct for this bias, we fit our model to additional data detailing the numbers of deaths due to HIV/AIDS from 1985 onwards, correcting for these two sources of biases.[4] We assume that the differences between the deaths recorded in the Mortality Information System and the estimates from Fazito *et al* are due to cases which have remained unreported and are classified as HIV/AIDS-related deaths retrospectively. The Brazilian Ministry of Health calculates that in 2007, 89.7% of deaths in Brazil were registered in the system. After adjusting for misclassification, Fazito *et al* suggest an underestimation of AIDS-related deaths between 13% in 2009 and 75% in 1986. We conservatively assume that the trends in mortality reporting in 2009 remain constant until the end of 2016. The more optimistic assumption that reporting trends continued to improve from 1999 until 2016 (estimated by fitting a linear model to the underreporting estimates and extrapolating forward to 2016), resulted in a significantly worse fit to the surveillance data (data not shown).

**Uncertainty analysis**

To examine the impact of uncertainty regarding the natural history of HIV on the incidence and epidemic trends presented here, we conducted a systematic multivariate uncertainty analysis by re-estimating all parameters under alternative sets of assumptions on the natural history input parameters. We considered eight scenarios for both male and female models to represent the variability in the input parameters relating to initial state probabilities, progression rates through CD4 states and mortality in ART-naïve individuals (Tables 3,4), corresponding with upper or lower limits derived from the interquartile ranges of the parameters. We used these narrow bounds as extreme values in these parameters yield implausible epidemic trends based on observed data. The joint posterior distributions were re-estimated under each of the eight scenarios using MCMC sampling and we present here the inferred incidence curves for each of the scenarios for both men and women (Figure S5). The median number of new infections estimated for 2015 varied between 32,000 – 52,000 for men and 13,000 – 20,000 for women, compared with 37,000 (28,000 – 54,000) infections in men and 16,000 (10,000 – 23,000) infections in women estimated in the main analysis. Epidemic trends inferred under alternative assumptions on HIV natural history yielded qualitatively similar results to our main findings, namely a double peak in the epidemic curve consistent with the data on observed cases.

We also varied the number of knots in the B-spline function to assess whether using a more rigid or a more flexible function would alter the shape of the incidence curve. The B-spline in the main analysis contained 10 knots, including 6 internal knots and four anchor points. Decreasing the number of internal knots did not substantially alter the epidemic curve (Figure S6) and resulted in an estimated 40,000 (95% CI 20,000 – 47,000) new infections in men and 16,000 (9,000 – 29,000) new infections in women in 2015. Using a less flexible function with 4 internal knots did not alter the timing of the peak and produced estimates of 43,000 (35,000 – 48,000) new infections in men and 25,000 (20,000 – 33,000) new infections in women in 2015. Both sets of models were compared to the models presented in the main analysis using the Bayesian Information Criterion which penalises for the numbers of parameters and this measure indicated a significantly poorer choice of model compared with the 10-knot B-spline model, which is used as the main set of results presented in this paper.

**Survival on ART**

We estimated mortality rates on ART directly from the integrated surveillance data in Brazil (Table S6). Individuals were included in the analysis if they were aged over 15 years at the time of reporting, started ART between 2006 and 2016, and had at least one CD4 count within 6 months prior to starting ART. We exclude those starting ART in 2016 due to insufficient follow-up time. Those individuals missing data on age or sex were also excluded from the analysis. The exposure time was calculated as the time from the date of the first ART prescription recorded until either a recorded death date or a censoring event. The censoring event was determined as the last record documented in any of the routine surveillance programmes.

A piecewise constant exponential regression model was used allowing the baseline hazard to vary between time periods to estimate changing mortality rates on ART by numbers of years on treatment (first year on ART, second year on ART onwards) and we included covariates on age-group, CD4 state prior to ART initiation and sex as fixed predictors. Individuals were classified into one of four CD4 states (≥ 500, 350-499, 200-349, < 200 cells/ μl) at ART initiation using the last CD4 count reported before starting ART. We assumed that mortality rates were constant within each age-group and CD4 state. Confidence intervals around the estimates were derived from the model assuming normality.

Of the 547,495 records of people initiating ART in Brazil, 277,803 individuals were eligible for inclusion in this analysis, 169,860 males and 107,880 females. All covariates (age-group, sex, calendar year and CD4 count at ART initiation) were tested using univariate analysis and were retained in the final model if they showed both a significant association with mortality and subsequently improved the fit of the multivariate model, assessed using the likelihood ratio test. ART data records in Brazil begin in 2006 and so we estimate the hazard ratios associated with calendar year pre-2006 by linear interpolation. The survival analysis does not consider non-adherence to ART treatment programmes, but estimates rates assuming an intention-to-continue treatment. Given that the model does not explicitly allow treatment drop-out, this generalised approach was more suitable, with our survival rates broadly reflecting the trends in adherence. We assume that those included in the analysis are representative of the population modelled here.

| Parameter |  |  | Value (range) | | | |
| --- | --- | --- | --- | --- | --- | --- |
|  |  | Sex | Age 15-24 | Age 25-34 | Age 35-44 | Age >45 |
| Initial state probabilities |  |  |  |  |  |  |
| CD4 ≥ 500 | *p_1_* | M | 0.547 | 0.503 | 0.455 | 0.380 |
|  |  | F | 0.592 | 0.547 | 0.499 | 0.420 |
| CD4 350-499 | *p_2_* | M | 0.246 | 0.246 | 0.250 | 0.247 |
|  |  | F | 0.209 | 0.217 | 0.221 | 0.221 |
| CD4 200-349 | *p_3_* | M | 0.159 | 0.188 | 0.219 | 0.267 |
|  |  | F | 0.142 | 0.169 | 0.199 | 0.245 |
| CD4 < 200 | *p_4_* | M | Calculated as 1-(p500 + p350 + p200) | | | |
|  |  | F | Calculated as 1-(p500 + p350 + p200) | | | |
| Duration in state (years) |  |  |  |  |  |  |
| CD4 ≥ 500 | *q_1_* | M | 5.04 | 5.04 | 5.04 | 5.04 |
|  |  | F | 5.45 | 5.45 | 5.45 | 5.45 |
| CD4 350-499 | *q_2_* | M | 3.04 | 3.04 | 3.04 | 3.04 |
|  |  | F | 3.28 | 3.28 | 3.28 | 3.28 |
| CD4 200-349 | *q_3_* | M | 4.94 | 4.94 | 4.94 | 4.94 |
|  |  | F | 5.33 | 5.33 | 5.33 | 5.33 |
|  |  |  |  |  |  |  |
| Annual AIDS-related mortality rates  (ART-naïve) |  |  |  |  |  |  |
| CD4 ≥ 500 | *µ_1_* | M | 0.003 | 0.004 | 0.005 | 0.007 |
|  |  | F | 0.003 | 0.004 | 0.005 | 0.007 |
| CD4 350-499 | *µ _2_* | M | 0.009 | 0.011 | 0.014 | 0.020 |
|  |  | F | 0.009 | 0.011 | 0.014 | 0.020 |
| CD4 200-349 | *µ _3_* | M | 0.009 | 0.012 | 0.015 | 0.021 |
|  |  | F | 0.010 | 0.012 | 0.015 | 0.022 |
| CD4 < 200 | *µ _4_* | M | 0.271 | 0.296 | 0.320 | 0.364 |
|  |  | F | 0.263 | 0.286 | 0.309 | 0.352 |

Table S1. Parameters relating to natural history of HIV infection, adapted from Mangal *et al*.[5] Note progression rates do not vary by age.

| Parameter | Description | Prior distribution  male model | Prior distribution  female model |
| --- | --- | --- | --- |
|  |  |  |  |
| *V_n_* | Internal knot position vector | $V_{1}\sim U\left( 1975-1985 \right)$ | $V_{1}\sim U\left( 1975-1985 \right)$ |
|  |  | $V_{2}\sim U\left( 1985-1995 \right)$ | $V_{2}\sim U\left( 1985-1995 \right)$ |
|  |  | $V_{3}\sim U\left( 1995-2000 \right)$ | $V_{3}\sim U\left( 1995-2000 \right)$ |
|  |  | $V_{4}\sim U\left( 2000-2005 \right)$ | $V_{4}\sim U\left( 2000-2005 \right)$ |
|  |  | $V_{5}\sim U\left( 2005-2010 \right)$ | $V_{5}\sim U\left( 2005-2010 \right)$ |
|  |  | $V_{6}\sim U\left( 2010-2016 \right)$ | $V_{6}\sim U\left( 2010-2016 \right)$ |
|  |  |  |  |
| *B_n_* | Basis coefficients  for spline function | $B_{1}=0$ | $B_{1}=0$ |
|  |  | $B_{2}\sim U\left( 1-60 \right)$ | $B_{2}\sim U\left( 1-60 \right)$ |
|  |  | $B_{n}\sim U\left( 5-5000 \right) for n=3,\ldots,6$ | $B_{n}\sim U\left( 5-3000 \right) for n=3,\ldots,6$ |
|  |  |  |  |
| *d_i_* | Monthly reporting rates  (baseline) | $d_{1}\sim\left\{ \begin{aligned} 0 t<1997 \\ U\left( 0,50 \right) t= 1997 \end{aligned} \right.$ | $d_{1}\sim\left\{ \begin{aligned} 0 t<1997 \\ U\left( 0,50 \right) t= 1997 \end{aligned} \right.$ |
|  |  | $d_{2}\sim\left\{ \begin{aligned} 0 t<1997 \\ U\left( 0,50 \right) t= 1997 \end{aligned} \right.$ | $d_{2}\sim\left\{ \begin{aligned} 0 t<1997 \\ U\left( 0,50 \right) t= 1997 \end{aligned} \right.$ |
|  |  | $d_{3}={0.01}^{a} t=1980$ | $d_{3}=0.01 t=1980$ |
|  |  | $d_{4}={0.05}^{a} t=1980$ | $d_{4}=0.05 t=1980$ |
|  |  |  |  |
| *ω_i_* | Incremental increase  in reporting rates  (per month) | $\omega_{1}=\left\{ \begin{aligned} 0 t<2001 \\ U\left( 0,50 \right) 2001\leq t\geq2009 \\ U\left( 0.5,50 \right) t\geq2010 \end{aligned} \right.$  $\omega_{2}=\left\{ \begin{aligned} 0 t<2001 \\ U\left( 0,50 \right) 2001\leq t\geq2009 \\ U\left( 0.5,50 \right) t\geq2010 \end{aligned} \right.$  $\omega_{3}=\left\{ \begin{aligned} U\left( 0,50 \right) t<2001 \\ U\left( 0,50 \right) 2001\leq t\geq2009 \\ U\left( 0.5,50 \right) t\geq2010 \end{aligned} \right.$  $\omega_{4}=\left\{ \begin{aligned} U\left( 0,50 \right) t<2001 \\ U\left( 0,50 \right) 2001\leq t\geq2009 \\ U\left( 0.5,50 \right) t\geq2010 \end{aligned} \right.$ | $\omega_{1}=\left\{ \begin{aligned} 0 t<2001 \\ U\left( 0,50 \right) 2001\leq t\geq2009 \\ U\left( 0.5,50 \right) t\geq2010 \end{aligned} \right.$  $\omega_{2}=\left\{ \begin{aligned} 0 t<2001 \\ U\left( 0,50 \right) 2001\leq t\geq2009 \\ U\left( 0.5,50 \right) t\geq2010 \end{aligned} \right.$  $\omega_{3}=\left\{ \begin{aligned} U\left( 0,50 \right) t<2001 \\ U\left( 0,50 \right) 2001\leq t\geq2009 \\ U\left( 0.5,50 \right) t\geq2010 \end{aligned} \right.$  $\omega_{4}=\left\{ \begin{aligned} U\left( 0,50 \right) t<2001 \\ U\left( 0,50 \right) 2001\leq t\geq2009 \\ U\left( 0.5,50 \right) t\geq2010 \end{aligned} \right.$ |
|  |  |  |  |
| *s_i_* | Monthly ART initiation rates  (baseline =1997) | $s_{1}\sim U\left( 0,5 \right)$  $s_{2}\sim U\left( 0,10 \right)$  $s_{3}\sim U\left( 0,10 \right)$  $s_{4}\sim U\left( 0,25 \right)$ | $s_{1}\sim U\left( 0,5 \right)$  $s_{2}\sim U\left( 0,10 \right)$  $s_{3}\sim U\left( 0,10 \right)$  $s_{4}\sim U\left( 0,25 \right)$ |
|  |  |  |  |
| *ε_i_* | Incremental increase  in ART initiation rates  (per year) | $\varepsilon_{1}\sim\left\{ \begin{aligned} U\left( 0,1 \right) 1998\leq t\geq2010 \\ U\left( 0,1 \right) 2011\leq t\geq2016 \end{aligned} \right.$ | $\varepsilon_{1}\sim\left\{ \begin{aligned} U\left( 0,1 \right) 1998\leq t\geq2010 \\ U\left( 0,1 \right) 2011\leq t\geq2016 \end{aligned} \right.$ |
|  |  | $\varepsilon_{2}\sim\left\{ \begin{aligned} U\left( 0,5 \right) 1998\leq t\geq2010 \\ U\left( 0,5 \right) 2011\leq t\geq2016 \end{aligned} \right.$ | $\varepsilon_{2}\sim\left\{ \begin{aligned} U\left( 0,5 \right) 1998\leq t\geq2010 \\ U\left( 0,5 \right) 2011\leq t\geq2016 \end{aligned} \right.$ |
|  |  | $\varepsilon_{3}\sim\left\{ \begin{aligned} U\left( 0,5 \right) 1998\leq t\geq2010 \\ U\left( 0,5 \right) 2011\leq t\geq2016 \end{aligned} \right.$ | $\varepsilon_{3}\sim\left\{ \begin{aligned} U\left( 0,5 \right) 1998\leq t\geq2010 \\ U\left( 0,5 \right) 2011\leq t\geq2016 \end{aligned} \right.$ |
|  |  | $\varepsilon_{4}\sim\left\{ \begin{aligned} U\left( 0,5 \right) 1998\leq t\geq2010 \\ U\left( 0,5 \right) 2011\leq t\geq2016 \end{aligned} \right.$ | $\varepsilon_{4}\sim\left\{ \begin{aligned} U\left( 0,5 \right) 1998\leq t\geq2010 \\ U\left( 0,5 \right) 2011\leq t\geq2016 \end{aligned} \right.$ |
|  |  |  |  |
| *α* | Age distribution parameters  baseline 1980-1990 | $\alpha\sim U\left( 0,1 \right)$ | $\alpha\sim U\left( 0,1 \right)$ |
| *β* |  | $\beta\sim U\left( 0,1 \right)$ | $\beta\sim U\left( 0,1 \right)$ |
| *γ* |  | $\gamma\sim U\left( 0,1 \right)$ | $\gamma\sim U\left( 0,1 \right)$ |
|  |  |  |  |
| *τ* | Adjustment in age distribution  parameters  (per year) | $\tau\sim U\left( -0.1,0.1 \right)$ | $\tau\sim U\left( -0.1,0.1 \right)$ |
| *υ* |  | $\upsilon\sim U\left( -0.1,0.1 \right)$ | $\upsilon\sim U\left( -0.1,0.1 \right)$ |
| *φ* |  | $\phi\sim U\left( -0.1,0.1 \right)$ | $\phi\sim U\left( -0.1,0.1 \right)$ |
|  |  |  |  |
| *m_d_* | Proportion of deaths reported  (detected cases) | $m_{d}\sim U\left( 0.5,1 \right)$ | $m_{d}\sim U\left( 0.5,1 \right)$ |
| *m_u_* | Proportion of deaths reported  (undetected cases) | $m_{u}\sim U\left( 0.5,1 \right)$ | $m_{u}\sim U\left( 0.5,1 \right)$ |
|  |  |  |  |
| *σ_md_* | Monthly adjustment in proportion  of deaths reported  (detected cases) from 2010 | $\sigma_{md}\sim U\left( 1\times{10}^{-5},1\times{10}^{-3} \right)$ | $\sigma_{md}\sim U\left( 1\times{10}^{-5},1\times{10}^{-3} \right)$ |
| *σ_mu_* | Monthly adjustment in proportion  of deaths reported  (undetected cases) from 2010 | $\sigma_{mu}\sim U\left( 1\times{10}^{-5},1\times{10}^{-3} \right)$ | $\sigma_{mu}\sim U\left( 1\times{10}^{-5},1\times{10}^{-3} \right)$ |

^a^ Values are assumptions

Table S2. Model parameters with associated prior distributions.

| Parameter | CD4 state | Males | | Females | |
| --- | --- | --- | --- | --- | --- |
|  |  |  | |  | |
| Initial state probabilities |  | lower | upper | lower | upper |
| Ages 15-24 years | ≥ 500 | 0.508 | 0.587 | 0.539 | 0.645 |
|  | 350-499 | 0.169 | 0.310 | 0.129 | 0.289 |
|  | 200-349 | 0.126 | 0.191 | 0.094 | 0.191 |
| Ages 25-34 years | ≥ 500 | 0.473 | 0.533 | 0.500 | 0.594 |
|  | 350-499 | 0.205 | 0.287 | 0.139 | 0.295 |
|  | 200-349 | 0.167 | 0.209 | 0.111 | 0.227 |
| Ages 35-44 years | ≥ 500 | 0.425 | 0.485 | 0.454 | 0.544 |
|  | 350-499 | 0.194 | 0.305 | 0.109 | 0.334 |
|  | 200-349 | 0.192 | 0.247 | 0.131 | 0.267 |
| Ages >45 years | ≥ 500 | 0.342 | 0.417 | 0.376 | 0.465 |
|  | 350-499 | 0.152 | 0.342 | 0.067 | 0.376 |
|  | 200-349 | 0.226 | 0.309 | 0.161 | 0.329 |
|  |  |  |  |  |  |
| Duration in CD4 state (years) |  |  |  |  |  |
| All ages | ≥ 500 | 4.32 | 5.86 | 4.70 | 6.28 |
|  | 350-499 | 2.50 | 3.76 | 2.68 | 4.07 |
|  | 200-349 | 3.45 | 6.94 | 3.73 | 7.40 |
|  |  |  |  |  |  |
| Annual AIDS-related mortality rates (ART-naïve) |  |  |  |  |  |
| Ages 15-24 years | ≥ 500 | 0.002 | 0.005 | 0.002 | 0.005 |
|  | 350-499 | 0.005 | 0.014 | 0.006 | 0.015 |
|  | 200-349 | 0.006 | 0.014 | 0.006 | 0.015 |
|  | < 200 | 0.242 | 0.311 | 0.234 | 0.303 |
| Ages 25-34 years | ≥ 500 | 0.002 | 0.006 | 0.002 | 0.007 |
|  | 350-499 | 0.007 | 0.018 | 0.007 | 0.019 |
|  | 200-349 | 0.007 | 0.019 | 0.008 | 0.019 |
|  | < 200 | 0.262 | 0.344 | 0.252 | 0.334 |
| Ages 35-44 years | ≥ 500 | 0.003 | 0.008 | 0.003 | 0.008 |
|  | 350-499 | 0.008 | 0.023 | 0.008 | 0.024 |
|  | 200-349 | 0.009 | 0.023 | 0.009 | 0.024 |
|  | < 200 | 0.280 | 0.377 | 0.270 | 0.366 |
| Ages >45 years | ≥ 500 | 0.004 | 0.012 | 0.004 | 0.012 |
|  | 350-499 | 0.011 | 0.033 | 0.012 | 0.034 |
|  | 200-349 | 0.013 | 0.033 | 0.013 | 0.035 |
|  | < 200 | 0.313 | 0.440 | 0.301 | 0.429 |

Table S3. The lower and upper limits for each of the rates included in the sensitivity analysis.

| Model | Alternative natural history assumptions | | | Estimated incidence 2015 | |
| --- | --- | --- | --- | --- | --- |
|  | Initial state probabilities | CD4 progression rates | AIDS-related mortality rates  (ART-naïve) | Male | Female |
| 1 | High | Fast | High | 33622 | 13088 |
| 2 | High | Fast | Low | 32424 | 13189 |
| 3 | High | Slow | High | 36880 | 16524 |
| 4 | High | Slow | Low | 43377 | 20039 |
| 5 | Low | Fast | High | 51716 | 13743 |
| 6 | Low | Fast | Low | 46387 | 14321 |
| 7 | Low | Slow | High | 31944 | 15225 |
| 8 | Low | Slow | Low | 33367 | 16513 |

Table S4. Details of the eight scenarios assumed for the sensitivity analysis using alternative natural history assumptions. See Table S3 for parameter values.

Figure S1. The distribution of CD4 cell counts within six months of reporting time (>500, 350-499, 200-349 and <200 cells/μl) for men (upper panel) and women (lower panel) between 2001 - 2015. Augmented data are not presented here.

**Reporting rates**

Figure S2. Estimated annual reporting rates for men (upper figure) and women (lower figure) for each CD4 state between 1980 and 2016.


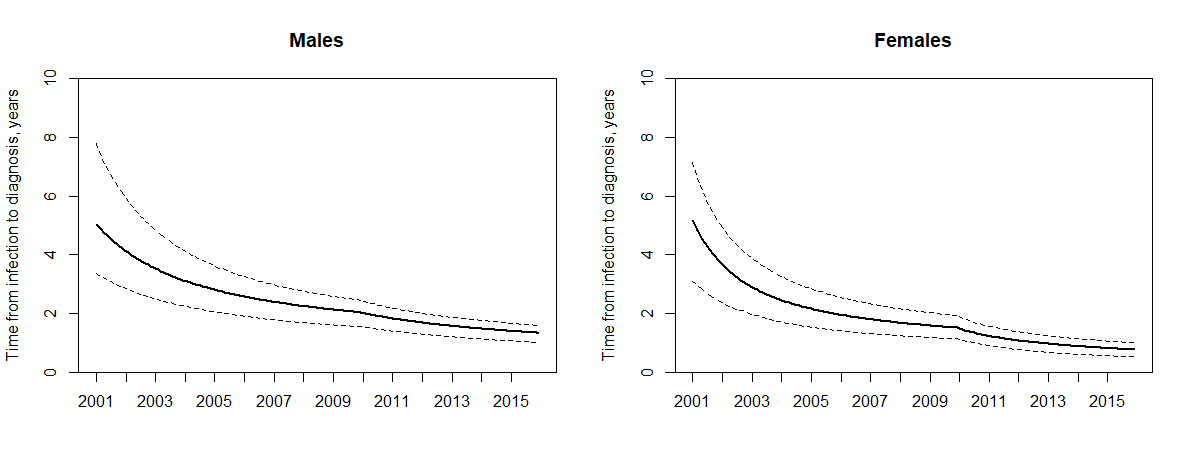
Figure S3. Mean time from infection to diagnosis for males (left) and females (right) aged between 15-24 years at infection.

**ART initiation rates**

Figure S4. Estimated annual rates of ART initiation for men (upper figure) and women (lower figure) for each CD4 state between 2006 and 2016.


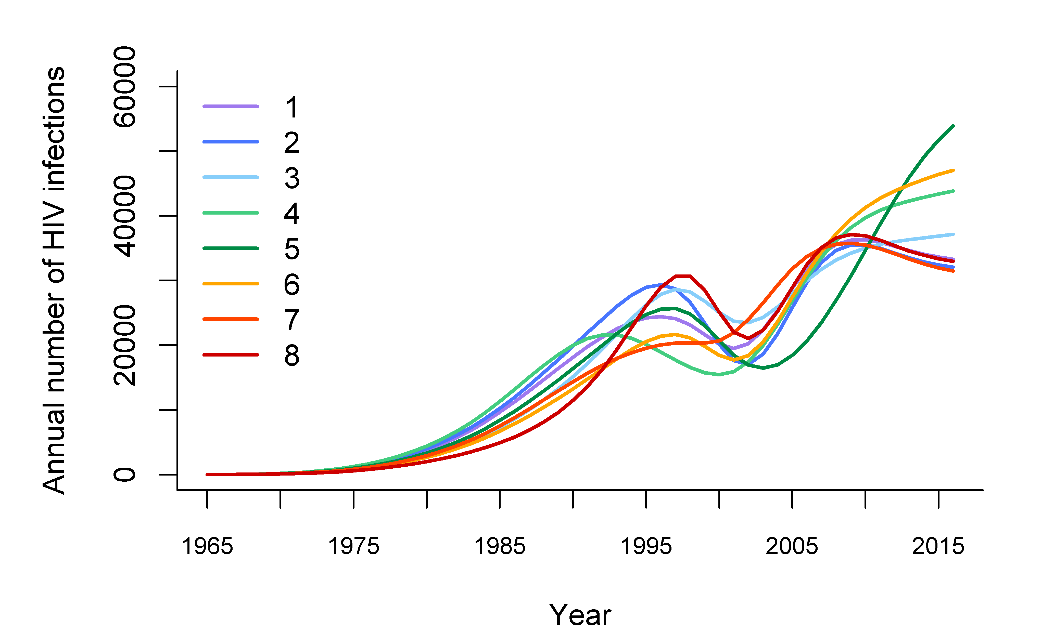


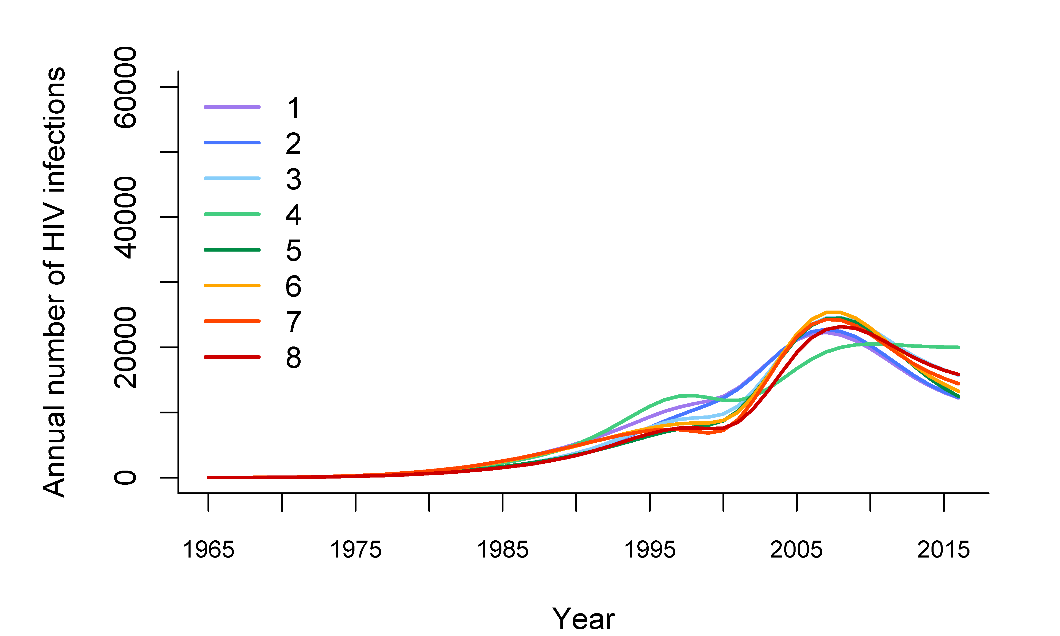


Figure S5. Inferred epidemic trends in HIV in males (upper figure) and females (lower figure) in Brazil under the alternative assumptions on the natural history of HIV infection – see Tables S2 and S3 for further details.


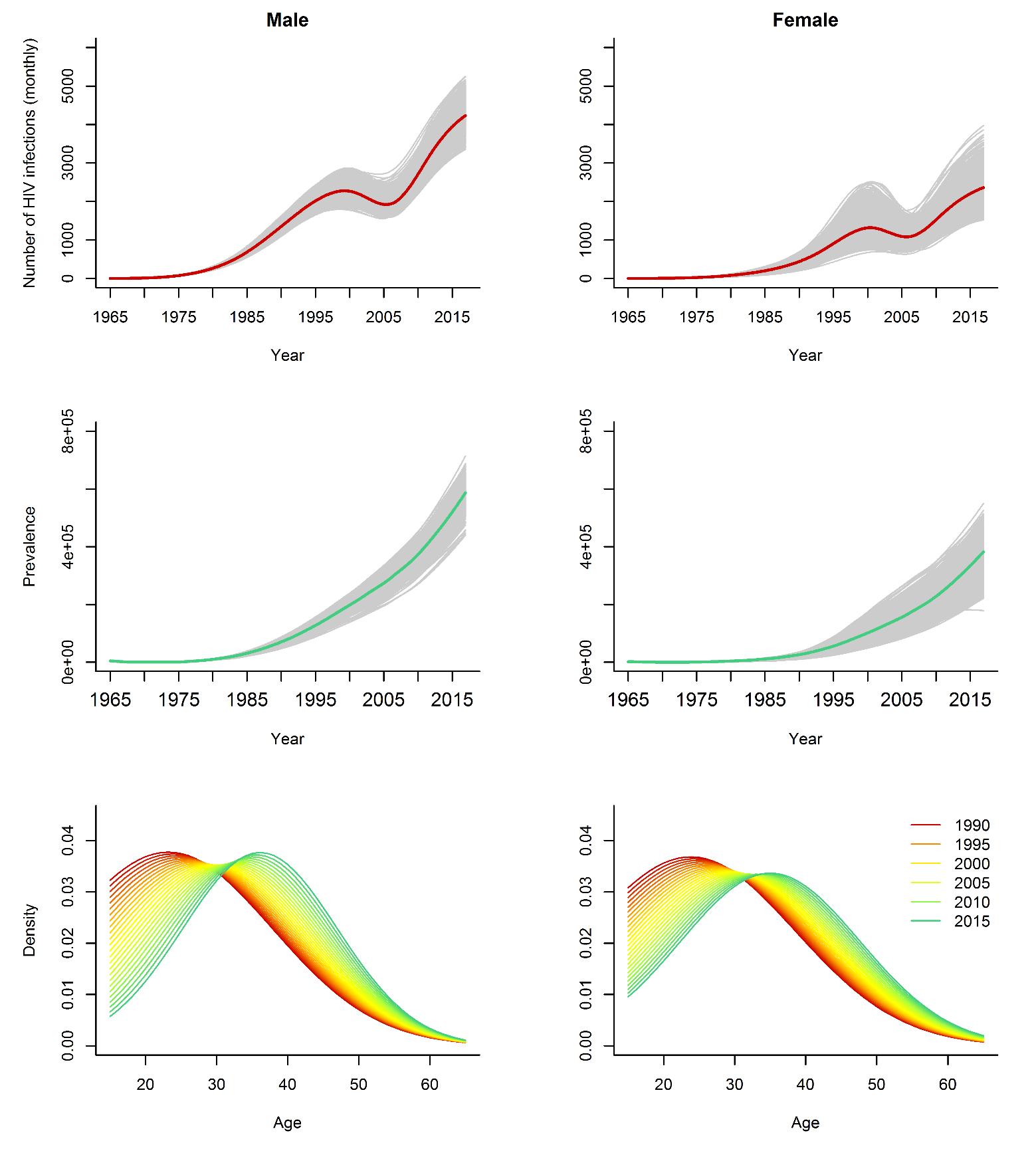


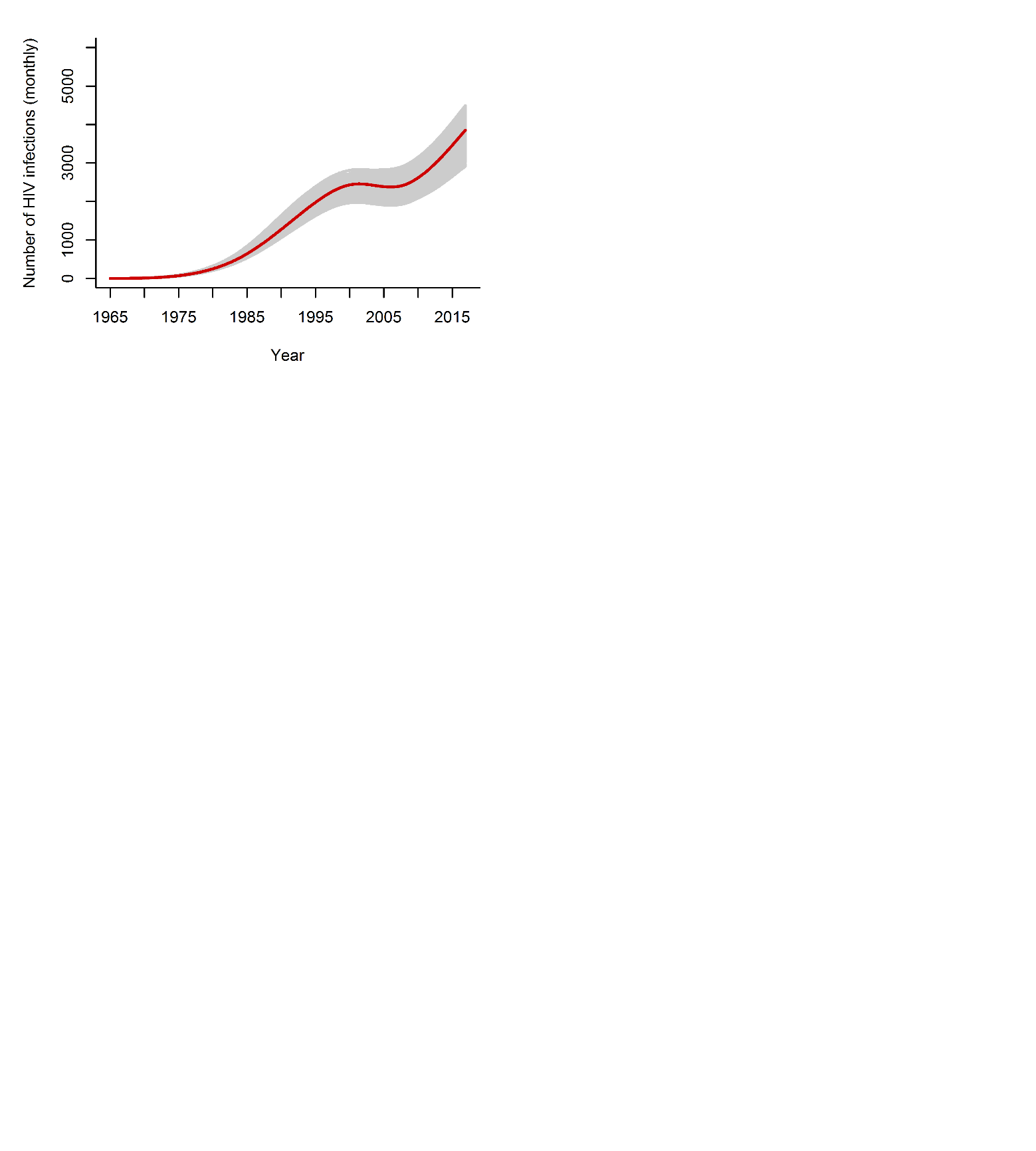

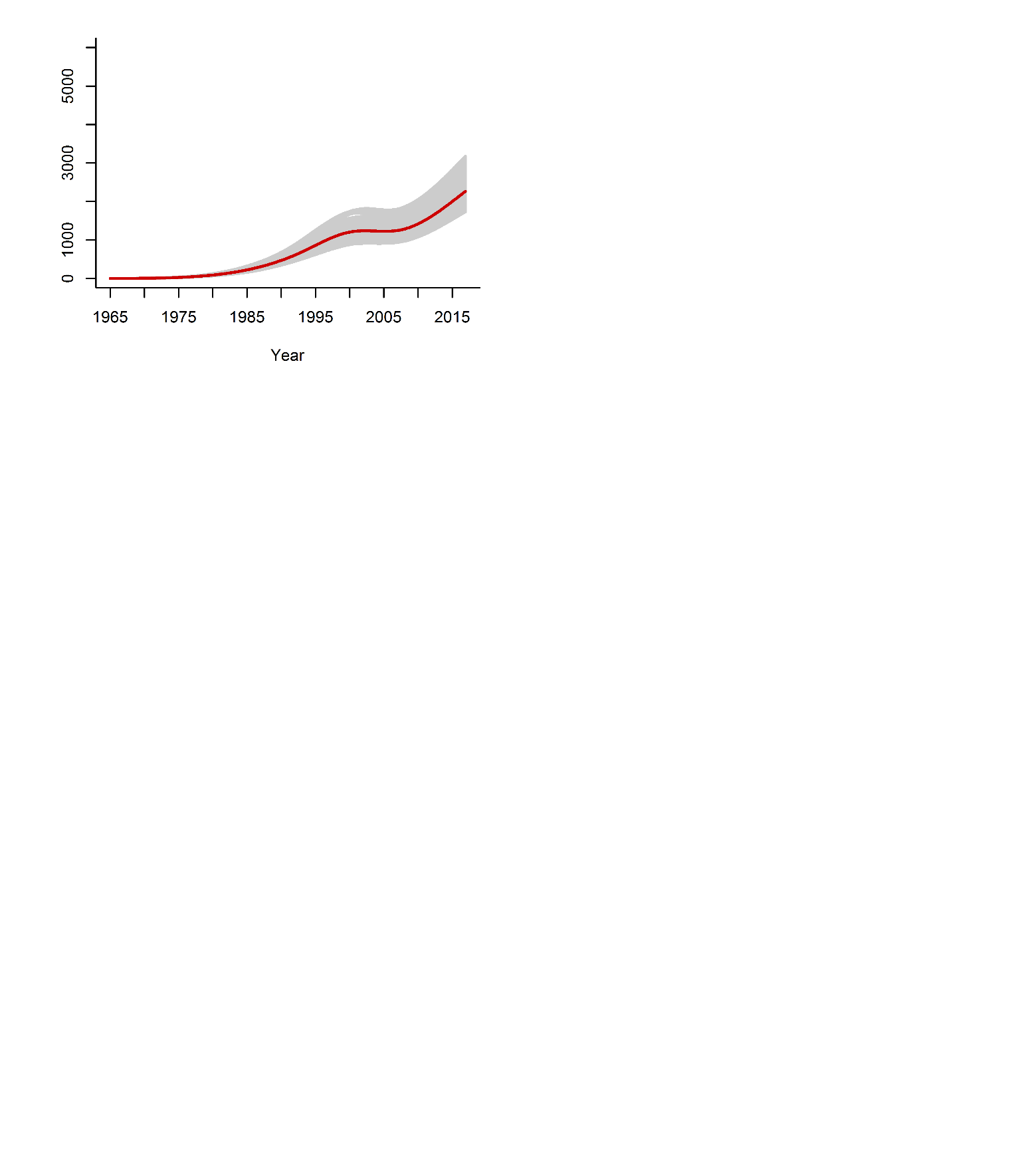


Figure S6. Sensitivity analysis adjusting the number of internal knots in the B-spline function. Estimated incidence trajectory using 5 internal knots (9 knots in total) in upper figures for men (left panels) and women (right panels) and using 4 internal knots (8 knots in total) lower figures.

|  | **Male** | | **Female** | |
| --- | --- | --- | --- | --- |
| **Spline function** | Log likelihood | BIC | Log likelihood | BIC |
| 6 knots* | -639756 | 1278950 | -364381 | 729324 |
| 5 knots | -639887 | 1280335 | -365674 | 731886 |
| 4 knots | -641235 | 1283032 | -367943 | 736423 |

Table S5. Log likelihood values and Bayesian information criterion (BIC) used for model selection. The BIC includes both a statistical value for the goodness of fit (log likelihood) and a penalty for increasing the number of parameters. Differences in BIC of over 10 indicate a significant difference between models and the lowest BIC is preferred.

* represents the model presented in the main analysis.

|  | Exponentiated coefficient | p-value |
| --- | --- | --- |
| Baseline hazard | 0.010  (0.009-0.010) | <0.0001 |
| Interval 1-12 months | 1.0 | -- |
| Interval >12 months | 0.40  (0.39-0.41) | <0.0001 |
|  |  |  |
| Age-group 15-24 | 1.0 | -- |
| Age-group 25-34 | 1.05  (1.00-1.10) | 0.07 |
| Age-group 35-44 | 1.16  (1.10-1.21) | <0.0001 |
| Age-group >45 | 1.42  (1.36-1.49) | <0.0001 |
|  |  |  |
| CD4 ≥ 500 | 1.0 | -- |
| CD4 350-499 | 1.45  (1.37-1.53) | <0.0001 |
| CD4 200-349 | 2.15  (2.06-2.26) | <0.0001 |
| CD4 < 200 | 5.85  (5.61-6.11) | <0.0001 |
|  |  |  |
| Sex F | 1.0 | -- |
| Sex M | 1.25  (1.22-1.28) | <0.0001 |
|  |  |  |
| Year 2006 | 1.0 | -- |
| Year 2007 | 1.04  (0.99-1.08) | 0.11 |
| Year 2008 | 0.98  (0.94-1.02) | 0.27 |
| Year 2009 | 0.93  (0.89-0.97) | 0.19 |
| Year 2010 | 0.87  (0.83-0.92) | 0.002 |
| Year 2011 | 0.81  (0.77-0.86) | <0.0001 |
| Year 2012 | 0.81  (0.76-0.85) | <0.0001 |
| Year 2013 | 0.72  (0.68-0.77) | <0.0001 |
| Year 2014 | 0.65  (0.61-0.69) | <0.0001 |
| Year 2015 | 0.52  (0.48-0.56) | <0.0001 |

Table S6. Estimated coefficients from a piecewise constant exponential regression model of survival on ART. Values are exponentiated coefficients representing annual baseline hazard rate and relative hazards for each covariate.

**REFERENCES**

1. Fonseca MGP, Coeli CM, Lucena FFA, Veloso VG, Carvalho MS. Accuracy of a probabilistic record linkage strategy applied to identify deaths among cases reported to the Brazilian AIDS surveillance database. *Cad Saude Publica* 2010,**26**:1431-1438.

2. United Nations Department of Economic and Social Affairs Population Division. World Population Prospects: The 2017 Revision, Key Findings and Advance Tables. In. Edited by United Nations. New York: United Nations; 2017.

3. Lodi S, Phillips A, Touloumi G, Geskus R, Meyer L, Thiébaut R*, et al.* Time From Human Immunodeficiency Virus Seroconversion to Reaching CD4+ Cell Count Thresholds <200, <350, and <500 Cells/mm3: Assessment of Need Following Changes in Treatment Guidelines. *Clinical Infectious Diseases* 2011,**53**:817-825.

4. Fazito E, Cuchi P, Ma Fat D, Ghys PD, Pereira MG, Vasconcelos AMN*, et al.* Identifying and quantifying misclassified and under-reported AIDS deaths in Brazil: a retrospective analysis from 1985 to 2009. *Sex Transm Infect* 2012,**88**:i86-i94.

5. Mangal TD. Joint estimation of CD4+ cell progression and survival in untreated individuals with HIV-1 infection. *AIDS (London, England)* 2017,**31**:1073.
